# Updating the Scientific Advisory Committee on Nutrition’s Framework for the evaluation of evidence

**DOI:** 10.1017/S0007114525104054

**Published:** 2025-08-14

**Authors:** Mamta Singh, Rachel Elsom, Adrienne Cullum, Russell J. de Souza, Darren C. Greenwood, David J. Mela, Ken K. Ong, Ian S. Young, Julie A. Lovegrove

**Affiliations:** 1 SACN Secretariat, Office for Health Improvement and Disparities (OHID), Department of Health and Social Care (DHSC), 39 Victoria Street, London SW1H 0EU, UK; 2 Members of SACN Subgroup on the Framework and Methods for the Evaluation of Evidence that Relates Foods and Nutrients to Health, OHID, DHSC, 39 Victoria Street, London SW1H 0EU, UK; 3 Chair of SACN Subgroup on the Framework and Methods for the Evaluation of Evidence that Relates Foods and Nutrients to Health, London, UK

**Keywords:** SACN, Framework, Evidence evaluation, Interpreting statistical data, Assessing evidence quality, Assessing evidence certainty

## Abstract

The Scientific Advisory Committee on Nutrition (SACN) provides independent advice on nutrition and related health matters to UK government organisations. In keeping with its commitment to openness and transparency, SACN follows a set ‘Framework’ to ensure a prescribed and consistent approach is taken in all its evidence evaluations. Following an update of the SACN Framework in 2020, which addressed some straightforward issues, the SACN Framework subgroup was established in 2021 to consider more complex matters that were not addressed in the 2020 update. The SACN Framework subgroup considered four main topics for update: (1) the different types of evidence evaluations produced by SACN, (2) interpretation of statistical data, (3) tools for assessment of study quality and (4) tools to assess the certainty of a body of evidence for exposure–outcome relationships. The Framework subgroup agreed clear definitions and processes for the different types of evidence evaluations produced by SACN and agreed that interpretation of *P* values should be informed by consideration of study size, power and methodological quality. The subgroup recommended use of the AMSTAR 2 tool for quality assessment of evidence from systematic reviews and use of the Grading of recommendations, assessment, development and evaluation approach to assess the certainty of evidence. The updated Framework was published in January 2023. This was followed by publication of a further update in October 2024. As a ‘living’ document, the Framework will be subject to regular review by the Framework subgroup and continue to evolve in line with best practice.

The Scientific Advisory Committee on Nutrition (SACN) is a committee of the UK Office for Health Improvement and Disparities. It provides independent scientific advice on nutrition and related health issues. It advises the governments of England, Scotland, Wales and Northern Ireland and is supported by a scientific secretariat based at UK Office for Health Improvement and Disparities. SACN was established in 2001, succeeding the Committee on Medical Aspects of Food and Nutrition Policy.

SACN’s remit is to assess the benefits and risks to health of nutrients, dietary patterns, food or food components and to make dietary recommendations for the UK population based on its assessment. SACN is committed to values of openness and transparency in recognition that these principles underpin public confidence in the scientific evaluation process. Where possible, meetings are held in open session except when ongoing evidence evaluations are being considered. This is to allow free discussion of the evidence and formulation of draft conclusions and recommendations before these are made available for public consultation or publication.

National and international risk assessment bodies such as the National Institute for Health and Care Excellence, the World Health Organization (WHO) and the European Food Safety Authority conduct or commission their own reviews of the primary evidence. However, SACN utilises existing published systematic reviews and meta-analyses, which may be supplemented by data on dietary intakes and nutritional status, and analyses and modelling of specific exposures. The benefits of SACN’s approach include making use of the existing evidence base and drawing on broader scientific expertise. A limitation is that the value of systematic reviews depends on their quality and the analyses conducted. In addition, the relevance and generalisability of the results of systematic reviews depend on how closely the systematic review question aligns with SACN’s research question, the UK population and how recent the review is.

To ensure a consistent prescribed approach, SACN follows a set ‘Framework’ to evaluate the evidence. The Framework provides SACN with an *a priori*, pre-determined set of methods for evaluating evidence. This guards against *ad hoc* or variable standards for evidence assessments. It also guards against individual or group bias because it specifies the types of evidence considered and their objective assessment. The Framework is a ‘living’ document, subject to regular review by a standing ‘SACN subgroup on the framework and methods for the evaluation of evidence that relates foods and nutrients to health’. This allows it to be modified and updated with version control as new methods are included.

At SACN’s first meeting in June 2001, the committee noted the requirement to be explicit about its approach to risk assessment. The initial SACN ‘Framework for evaluation of evidence’ was published and adopted as a working document in June 2002. It was reviewed in 2003 and 2008, but no amendments were made. An updated Framework was published in 2012, reflecting how SACN’s approach to risk assessment had evolved since the Framework was originally devised. A broad range of issues for potential update were subsequently identified at SACN meetings in November 2018 and March 2019. A ‘refreshed’ Framework addressing the straightforward issues was published in March 2020.

The SACN subgroup on the framework and methods for the evaluation of evidence that relates foods and nutrients to health (hereafter referred to as the subgroup) was established at the SACN March 2021 meeting to consider the more complex issues that were not addressed in the 2020 refresh. The subgroup’s role is to provide ongoing methodological support to SACN, its working groups and the subgroup on maternal and child nutrition. It also ensures the SACN Framework remains under review and continues to be fit for purpose. Details of the subgroup’s terms of reference, membership and minutes of meetings are available to view on the SACN website. An updated ‘Framework and methods for the evaluation of evidence that relates foods and nutrient to health’ was published on the SACN website in January 2023 and updated again in October 2024 (version 2024/01)^(1)^. Earlier versions of the Framework are also available to view.

This paper summarises the process and approach taken by SACN to update the Framework and formalise the use of contemporary tools for evidence-based risk assessment.

## Methods

At the first meeting of the subgroup in May 2021, the following four areas were prioritised for consideration: (i) the different types of evidence evaluations produced by SACN; (ii) interpretation of statistical methods and data; (iii) tools to assess study quality and (iv) tools to assess the certainty of exposure-outcome relationships. These topics were considered in depth over the course of the next four meetings. The most extensive and detailed considerations related to assessing the certainty of evidence.

Following the subgroup’s revisions, the draft updated Framework was considered by SACN members, then amended to take account of their comments.

## Results

The subgroup’s considerations and decisions relating to the four priority topics are summarised below.

### Scientific Advisory Committee on Nutrition’s evidence evaluations

Since its inception in 2001, SACN has produced a range of publications. The different approaches and nomenclature have changed over time, reflecting the need for flexibility in the types of evaluations undertaken by SACN. The following approaches, with clear definitions and processes, were agreed for consistency in future evidence evaluations: reports (full risk assessments); rapid reviews; position statements; updates to reports/rapid reviews/position statements and joint reports/rapid reviews/position statements (to cover assessments jointly undertaken with other scientific committees).

It was agreed that the appropriate approach should be chosen at the outset of an evaluation, with the rationale for the selection included in the methods section. The choice of approach would depend on consideration of issues such as the research question(s), the nature of the available evidence and the urgency and timeframe for completion.

### Interpretation of statistical methods and data

In previous SACN reports, findings with a *P* value < 0·05 were considered ‘statistically significant’ and providing evidence of an effect (from randomised controlled trials) or association (from observational studies), while those with a *P* value ≥ 0·05 were considered as ‘not statistically significant’ and providing insufficient evidence of an effect or association. During the preparation of more recent reports, SACN members had raised concerns about using the *P*< 0·05 criterion alone for interpretation of results. Studies with *P* values just below 0·05 might also be at greater risk of publication bias because those with ‘statistically significant’ results are more likely to be published^(2)^.

The subgroup agreed that *P* values should not be considered in isolation and that it would be more informative to also consider effect size and confidence intervals. Clinical or biological significance and public health relevance should also be considered since findings might be ‘statistically significant’ but may not be clinically or biologically important. It was also noted that a small effect size may have little value at an individual level but could be important at a population level.

For interpretation of study results, it would be important to define outcomes and the effect sizes considered beneficial for public health at the outset of an evidence evaluation. Interpretation of *P* values should be informed by consideration of study size and power. It would also be essential to consider the methodological quality of studies because, irrespective of the *P* value, findings from poor quality studies or studies with a high risk of confounding may not be reliable. The subgroup agreed that future evidence evaluations should not describe results as ‘significant’ or ‘non-significant’ but report the exact *P* value, estimated effect size and confidence intervals where available, alongside the direction of any effect or association and consistency of findings.

### Assessing the quality of evidence

#### General approach

Assessment of evidence quality in SACN reports was previously based on criteria specified in the SACN Framework. The subgroup agreed that future evidence evaluations should, where possible, use externally developed and recognised quality assessment tools.

#### Assessing systematic reviews

Since the majority of SACN’s assessments are based on evidence from systematic reviews, two established quality assessment tools for systematic reviews and meta-analyses were considered: AMSTAR 2 (a measurement tool to assess systematic reviews)^(3)^ and ROBIS (risk of bias in systematic reviews)^(4)^. The usability in practice of AMSTAR 2 and ROBIS was compared by applying both to assess the quality of two systematic reviews with meta-analyses (Korsmo-Haugen et al.^(5)^, Sainsbury et al.^(6)^) that were previously reviewed in the SACN report on Lower carbohydrate diets for adults with type 2 diabetes^(7)^.

The AMSTAR 2 tool assesses methodological quality through a checklist of sixteen questions. Seven of the questions are considered ‘critical’ to the validity and conclusions of the systematic review (although appraisers may add or substitute other critical domains). Responses for eleven questions are dichotomous (*yes/no*), while responses for five questions include an additional response (*partial yes)*. An overall judgement of confidence (*high*, *moderate*, *low* or *very low*) in the results of a systematic review is based on the assessment of the critical and non-critical items. The AMSTAR 2 assessments of the systematic reviews with meta-analyses by Korsmo-Haugen et al.^(5)^ and Sainsbury et al.^(6)^ are summarised in online Supplementary Table 1.

The ROBIS tool assesses risk of bias in three phases. The 1st phase assesses relevance of the systematic review to the research question of interest by comparing both in terms of participants, interventions, comparisons and outcomes. The 2nd phase identifies concerns within the systematic review process and comprises twenty-one questions within four domains (study eligibility criteria, identification and selection of studies; data collection and study appraisal; synthesis and findings). There are five possible responses to the questions (*yes*, *probably yes*, *probably no*, *no* or *no information*). The 3rd phase comprises three questions and considers if the systematic review, as a whole, is at risk of bias. A judgement is then made on the overall risk of bias (*low*, *high* or *unclear*).

In the 1st phase of the ROBIS assessments (relevance to research question) both Korsmo-Haugen et al.^(5)^ and Sainsbury et al.^(6)^ were considered relevant to the research question but Sainsbury et al.^(6)^ was judged to be a partial match since it addressed only two of the three outcomes of interest. The concerns identified within the systematic review process (phase 2) are summarised in online Supplementary Table 2, and judgement on the overall risk of bias (phase 3) is provided in online Supplementary Table 3.

The overall judgements on the quality of the 2 systematic reviews using AMSTAR 2 and ROBIS were in agreement. For the systematic review by Korsmo-Haugen et al.^(5)^, overall confidence in the results was ‘high’ using AMSTAR 2, and risk of bias was ‘low’ using ROBIS; for the systematic review by Sainsbury et al.^(6)^, overall confidence in results was ‘low’ using AMSTAR 2, and risk of bias was ‘high’ using ROBIS.

The subgroup agreed that both AMSTAR 2 and ROBIS provided a structured approach to assess the quality of the systematic reviews. Overall, AMSTAR 2 was simpler and easier to use. ROBIS provided a more rigorous assessment of risk of bias and consequently took much more time to complete. The longer completion time was identified as potentially problematic if the quality of several systematic reviews needed to be assessed for a SACN evidence evaluation. The main advantages and disadvantages of the AMSTAR 2 and ROBIS tools, experienced during the comparison exercise, are summarised in Table 1.

**Table 1.** Advantages and disadvantages of AMSTAR 2^(3)^ and ROBIS^(4)^

	AMSTAR 2^(3)^	ROBIS^(4)^
Advantages	User friendly Relatively simple and straightforward to complete 2 or 3 responses to questions in checklist Flexibility in the assignment of critical domains	Detailed and thorough assessment of risk of bias Transparent – includes space to record rationale for responses to questions and for rating the level of concern
Disadvantages	Risk of bias not considered in great detail Does not include space to explain rationale for responses to checklist questions Does not allow the option of partial yes response for some questions with only yes/no response options	Requires expertise in subject content and systematic review methodology Lengthy, complex and difficult to complete Time consuming Some questions were difficult to assess 5 possible responses to questions; not always possible to distinguish between yes/probably yes and no/probably no No ‘moderate’ category for level of concern in each domain of phase 2 or in the overall risk of bias category in phase 3

The subgroup agreed to recommend use of the AMSTAR 2 tool for quality assessment of evidence from systematic reviews. It was agreed, however, to further explore the practicality of using ROBIS in the future.

#### Assessing primary research

For quality assessment of primary studies (when evidence from systematic reviews is not available), it was agreed that working groups should use ROB 2^(8)^ (revised Cochrane risk of bias tool for randomised trials) and ROBINS-I^(9)^ (risk of bias in non-randomised studies - of interventions) as appropriate.

#### Assessing guidelines from other organisations

The subgroup recognised that it would be appropriate and efficient for SACN’s evidence evaluations to be informed by reports or guidelines from relevant expert bodies (such as the WHO) that have systematically considered the evidence but have not been published in peer-reviewed journals. The AGREE II (Appraisal of Guidelines for Research and Evaluation) Instrument^(10)^ was identified and considered for the purpose of assessing the quality of guidelines. AGREE II comprises 23 items, grouped under 6 domains that consider different aspects of guideline quality: (1) scope and purpose; (2) stakeholder involvement; (3) rigour of development; (4) clarity of presentation; (5) applicability; and (6) editorial independence.

It was agreed to recommend use of the AGREE II tool for quality assessment of published reports and guidelines from relevant organisations. Domains considered to be particularly relevant to SACN’s evaluations were 1, 3 and 6. It was agreed that working groups should decide on the relevant domains (and items within these) at the outset of an evidence evaluation and describe these in the protocol.

### Assessing the certainty of evidence

The rationale for assessing the certainty of a body of evidence is to inform and guide recommendations. Previous versions of the SACN Framework did not include guidance for assessing evidence certainty. However, evidence was graded in 4 SACN reports using an approach devised by SACN. The approach was conceived initially for use in the SACN report on Carbohydrates and health^(11)^. It was developed for use in the SACN report on Saturated fats and health^(12)^ and then further developed for the SACN reports on Lower carbohydrate diets for adults with type 2 diabetes^(7)^ and Feeding young children aged 1–5 years^(13)^.

The subgroup agreed that a consistent and standardised approach, with wide international recognition and comparability, was required for assessing the certainty of evidence in future SACN evidence evaluations. Four approaches were considered: Grading of recommendations, assessment, development and evaluation (GRADE); Nutrigrade^(14)^; United States Department of Agriculture (USDA) Dietary Guidelines Advisory Committee (DGAC)^(15)^ and Hierarchies of Evidence Applied to Lifestyle Medicine (HEALM)^(16)^. Nutrigrade and HEALM required detailed evaluation of individual studies. Since SACN usually considers evidence from published systematic reviews, these approaches were not considered further. It was agreed to focus on the GRADE approach because it is the most recognised and widely used tool for assessing evidence certainty. It specifies 4 levels of certainty (*high*, *moderate*, *low* and *very low*) that can be assigned to a body of evidence per outcome (see Table 2).

**Table 2.** GRADE certainty ratings

Certainty	Interpretation
High	Very confident that the true effect lies close to that of the estimate of the effect
Moderate	Moderately confident in the effect estimate: the true effect is likely to be close to the estimate of the effect, but there is a possibility that it is substantially different
Low	Confidence in the effect estimate is limited: the true effect may be substantially different from the estimate of the effect
Very low	Very little confidence in the effect estimate: the true effect is likely to be substantially different from the estimate of effect

Evidence from randomised trials starts with a ‘high’ certainty rating. This can then be downgraded after considering 5 criteria: risk of bias; imprecision; inconsistency; indirectness; and publication bias. Evidence from observational studies usually starts with a ‘low’ certainty rating because of potential bias due to lack of randomisation and because confounding is always a concern in even the most rigorously conducted observational studies. The certainty rating from observational evidence can be upgraded if any of 3 criteria are met: (1) large magnitude of effect; (2) clear dose-response gradient; (3) residual confounding is likely to decrease rather than increase the magnitude of effect.

The subgroup agreed that the advantages of the GRADE approach included consistency, transparency and comparability with other guidelines. It also allowed flexibility to exercise judgements in the 5 key domains. However, GRADE presented specific challenges for assessing nutritional evidence. These included: (1) likelihood of ‘low’ certainty being assigned to macronutrient and whole-diet intervention trials, where blinding is impossible or unrealistic; (2) potentially undervaluing evidence from prospective cohort studies (which provide evidence of long-term effects in real life conditions) because the starting point for all observational evidence (regardless of type) is ‘low’ certainty; and (3) limited flexibility to upgrade evidence from observational studies.

Implementation of the GRADE approach in practice was explored by conducting a grading exercise, comparing it with the USDA/DGAC approach. Both approaches were applied to grade the evidence for the effect of lower compared to higher carbohydrate diets on an outcome considered in the SACN report on ‘Lower carbohydrate diets for adults with type 2 diabetes’^(7)^. The primary studies in all the systematic reviews considered in this report were randomised controlled trials. The outcome considered in the grading exercise was glycated haemoglobin (HbA1c) concentration in the longer term (≥12 months), in a systematic review with meta-analysis by Sainsbury et al.^(6)^.

Using GRADE, the evidence for HbA1c (≥ 12 months) was graded as: **moderate certainty of no difference in effect**. The process for reaching this grade is summarised in online Supplementary Table 4.

With the USDA/DGAC approach, a grade (*strong*, *moderate*, *limited*, *grade not assignable*) is assigned for each of 5 elements: risk of bias, consistency, directness, precision, and generalisability. The final grade reflects consideration of all the grading criteria. Using this approach, the evidence for HbA1c (≥ 12 months) was also graded as: **moderate certainty of no difference in effect**. The process for reaching this grade is summarised in online Supplementary Table 5.

In practice, the GRADE approach was considered to be more straightforward to use than the USDA/DGAC approach. The stepwise process for reaching a final grade was transparent and the reasons for downgrading were clear. With the USDA/DGAC approach, each of 5 assessment domains were assigned a grade but no guidance was provided on weighting the separate domains to make a judgement on the overall grade. Another concern was that the grades could inflate confidence in the evidence because there was no ‘weak’ or ‘low’ category.

Although the grading exercise generally favoured the GRADE approach, there were still concerns about the criteria for upgrading observational evidence. GRADE stipulates that observational evidence can be upgraded if there is a large magnitude of effect (risk ratio > 2 or < 0·5); however, such large effect sizes are rarely observed in nutrition evidence and a smaller effect size could be important in terms of public health. It was suggested that at the outset of an evidence evaluation, SACN working groups should decide and then specify the magnitude of effect that would be considered ‘large’ for each outcome under consideration. The agreed threshold could then be used as the basis to make a judgement about upgrading evidence from prospective cohort studies.

The secretariat subsequently met with two representatives of the GRADE Public Health Group to discuss interpretation of GRADE in relation to public health and nutrition evidence. The GRADE representatives agreed it would be appropriate for SACN working groups to set the threshold for a ‘large’ effect size (with justification provided) for a particular outcome. They also advised that any suggestions on making GRADE more usable for public health would be considered by the GRADE developers.

A remaining concern about adopting GRADE was its appropriateness in a nutrition context, where there is a paucity of evidence from large long-term randomised controlled trials or where allocation of intervention is masked from the participants. Instead, evidence for longer term hard endpoints is largely drawn from observational studies where grading starts at ‘low’ certainty.

The previous grading exercise, comparing the GRADE and USDA/DGAC approaches, was extended to a meta-analysis of prospective cohort studies^(17)^ that considered the association between sugar-sweetened beverage consumption in children aged 1–5 years and body weight in later childhood (5 prospective cohort studies/7 comparisons, *n* 7255). Comparison of higher *v*. lower sugar-sweetened beverage intakes suggested a higher risk of being overweight associated with higher sugar-sweetened beverage intakes (odds ratio 1·55, 95 % confidence interval 1·32 to 1·82, *P*< 0·001). The certainty of the evidence was assessed as ‘low’ using the GRADE approach and as ‘limited’ using the USDA/DGAC approach.

In common with the previous grading exercise (comparing both approaches to grade a meta-analysis of randomised controlled trials), applying the stepwise approach of GRADE was found to be more straightforward. The USDA/DGAC approach involved making separate judgements on each domain and seemed more subjective. It was noted that both approaches had reached a similar grade but that the process was more transparent with GRADE.

The subgroup agreed that, in general, SACN’s public health recommendations should be based on evidence assessed as ‘high’ or ‘moderate’ certainty. In some cases, however, expert judgement could also be used to make recommendations based on ‘low’ certainty providing that a clear explanation of the rationale for such a decision was included.

Overall, it was agreed that advantages of GRADE included its transparency, broader international recognition and its comparability. In addition, the GRADE developers were open to evolving the methods in response to feedback. Although the subgroup still had some reservations about applying GRADE to nutritional evidence, it was agreed to recommend its use in future SACN evidence evaluations. Since the updated Framework was intended to be a ‘live’ document, the approach to grade the certainty of evidence could be changed if GRADE was found to be unsuitable in practice.

The subgroup agreed that it would be important for the Framework to clearly describe the two-step process of (1) assessing evidence quality (applied at the systematic review level) and (2) grading evidence certainty (applied at the outcome level).

The updated Framework was published on the SACN website in January 2023. It was further updated in October 2024 to include more information on how SACN determines its work programme; recommendation to use the ROBINS-E^(18)^ (risk of bias in non-randomised studies – of exposures) tool to assess the quality of observational cohort studies; addition of text stating that (exceptionally) recommendations could be based on ‘very low’ certainty of evidence and a flow diagram illustrating the process for selecting systematic review/meta-analysis to grade evidence for an exposure–outcome relationship. The changes to the previous version were chronicled in an Annex to the Framework.

### Next steps

Since publication of the updated Framework, the subgroup has provided support and guidance to the SACN Nutrition and maternal health working group on applying GRADE to assess the certainty of evidence for the draft SACN report on nutrition and maternal weight outcomes. The subgroup has also considered and made recommendations to SACN for improving the consistency and clarity of terminology to express energy intakes and recommendations for fat and carbohydrate intakes. A number of topics have been identified for future consideration.

As a ‘living’ document, the Framework will be subject to regular review by the subgroup and continue to evolve in line with best practice. The subgroup will respond to any issues experienced by SACN, the subgroup on maternal and child nutrition or its working groups in applying the Framework to ongoing evidence evaluations.

The subgroup would welcome and consider any comments or feedback from the scientific community on the SACN Framework.

## Supporting information

Singh et al. supplementary material Singh et al. supplementary material

## Supplementary material

For supplementary material/s referred to in this article, please visit https://doi.org/10.1017/S0007114525104054
