# Updating the Scientific Advisory Committee on Nutrition’s Framework for the evaluation of evidence – CORRIGENDUM

**DOI:** 10.1017/S0007114525105060

**Published:** 2025-08-28

**Authors:** Mamta Singh, Rachel Elsom, Adrienne Cullum, Russell J. de Souza, Darren C. Greenwood, David J. Mela, Ken K. Ong, Ian S. Young, Julie A. Lovegrove

**Explanation of correction:** The above article was missing the full name of one of the authors, this has been updated in the original article.

**Original text and correction:**

**ORIGINAL TEXT**

Mamta Singh, Rachel Elsom, Adrienne Cullum, Russ J. de Souza, Darren C. Greenwood, David J. Mela, Ken K. Ong, Ian S. Young and Julie A. Lovegrove on behalf of the Scientific Advisory Committee on Nutrition (SACN)

**CORRECTION**

Mamta Singh, Rachel Elsom, Adrienne Cullum, Russell J. de Souza, Darren C. Greenwood, David J. Mela, Ken K. Ong, Ian S. Young and Julie A. Lovegrove on behalf of the Scientific Advisory Committee on Nutrition (SACN)
