# Supplementary material for: Updating the Scientific Advisory Committee on Nutrition’s Framework for the evaluation of evidence
Source: Br J Nutr. 2025 Jul 31;134(3):257–62. doi: 10.1017/S0007114525104054 (PMC12491393; doi:10.1017/S0007114525104054)
Supplement: Singh et al. supplementary material [file S0007114525104054sup001.docx]

**Supplementary Table S1** AMSTAR 2^(3)^ assessment of systematic reviews by Korsmo Haugen et al and Sainsbury et al

| **Criteria for evaluation** | Korsmo-Haugen et al^(5)^ | Sainsbury et al^(6)^ |
| --- | --- | --- |
| 1. Did the research questions and inclusion criteria for the review include the components of PICO? | yes | yes |
| 1. Did the report of the review contain an explicit statement that review methods were established prior to conduct of review and did the report justify any significant deviations from the protocol? | yes | yes |
| 1. Did the review authors explain their selection of the study designs for inclusion in the review? | not applicable | not applicable |
| 1. Did the review authors use a comprehensive literature search strategy? | partial yes | yes |
| 1. Did the review authors perform study selection in duplicate? | yes | yes |
| 1. Did the review authors perform data extraction in duplicate? | yes | yes |
| 1. Did the review authors provide a list of excluded studies and justify the exclusions? | yes | no |
| 1. Did review authors describe the included studies in adequate detail? | yes | yes |
| 1. Did the review authors use a satisfactory technique for assessing risk of bias in individual studies that were included in the review? | yes | yes |
| 1. Did the review authors report on the sources of funding for the studies included in the review? | no | yes |
| 1. If meta-analysis was performed, did the review authors use appropriate methods for statistical combination of results? | yes | yes |
| 1. If meta-analysis was performed, did the review authors assess the potential impact of risk of bias in individual studies on results of the meta-analysis or other evidence synthesis? | yes | yes |
| 1. Did the review authors account for risk of bias in primary studies when interpreting/discussing results of the review? | yes | yes |
| 1. Did the review authors provide a satisfactory explanation for, and discussion of, any heterogeneity observed in results of the review? | yes | yes |
| 1. If they performed quantitative synthesis (1) did the review authors carry out an adequate investigation of publication bias (small study bias) and (2) discuss its likely impact on results of review? | yes | partial yes* |
| 1. Did the review authors report any potential sources of conflict of interest, including any funding they received for conducting the review? | yes | yes |
| **Overall confidence in results** | **High**  *(1 non- critical weakness)* | **Low**  *(1 critical weakness)* |

Items 2, 4, 7, 9, 11, 13, 15 are considered critical domains

**partial yes* is not an option for this item but was used here because answer was ‘yes’ to point (1) and ‘no’ to point (2)

Supplementary Table 2 ROBIS^(4)^ phase 2 assessment of systematic reviews by Korsmo Haugen et al and Sainsbury et al: Summary of concerns and rationale

| **Domain** | Korsmo-Haugen et al^(5)^ | Sainsbury et al^(6)^ |
| --- | --- | --- |
| Concerns regarding specification of study eligibility criteria | **LOW** – Answers to all questions *yes* or *probably yes*, so no potential concerns about specification of eligibility criteria were identified.  Adhered to predefined objectives and eligibility criteria. However, justification for date restriction was not provided and criteria for type 2 diabetes diagnosis not specified. | **HIGH** - Potential concern that review did not adhere to predefined objectives and eligibility criteria. Change to predefined objectives and eligibility criteria not mentioned or justified.  Language restrictions mean potential risk that relevant studies were not included |
| Concerns regarding methods used to identify and/or select studies | **HIGH -** Answers to all but 1 question, *yes* or *probably yes.*  Did not provide rationale for date restriction for study search.  Language restrictions for study selection mean potential risk that relevant studies were not included. | **HIGH** - Answers to all but 1 question, *yes*.  Date restrictions appropriate but language restrictions mean potential risk that relevant studies were not included |
| Concerns regarding methods used to collect data and appraise studies | **LOW** - All questions rated as *yes* or *probably* *yes,* so no potential areas of bias identified. The review processes of data collection and study appraisal are therefore unlikely to have introduced bias into this systematic review. | **LOW -** All questions rated as *yes* or *probably yes,* so no potential areas of bias identified. The review processes of data collection and study appraisal are therefore unlikely to have introduced bias into this systematic review. |
| Concerns regarding synthesis and findings | **LOW** - All questions rated as *yes* or *probably yes,* so no potential concerns regarding synthesis and findings. Authors addressed heterogeneity in their analysis and explored using subgroup analyses. Risk of bias of individual studies was addressed and included as a subgroup analysis. | **LOW** - All questions rated as *yes* or *probably yes,* so no potential concerns regarding synthesis and findings. Heterogeneity was low; where moderate heterogeneity was detected, a sensitivity analysis was conducted. Risk of bias of individual studies was considered and addressed by conducting sensitivity analyses. |

Supplementary Table 3 ROBIS^(4)^ phase 3 assessment of systematic reviews by Korsmo Haugen et al and Sainsbury et al: Judging overall risk of bias

| **Signalling question** | Korsmo-Haugen et al^(5)^ | Sainsbury et al^(6)^ |
| --- | --- | --- |
| Did the interpretation of findings address all concerns identified in Domains 1 to 4? | no | no |
| Was the relevance of identified studies to the review's research question appropriately considered? | yes | yes |
| Did the reviewers avoid emphasising results on the basis of their statistical significance? | yes | yes |
| **Risk of bias in review** | **LOW** | **HIGH** |
| **Rationale** | Although potential risk that some studies were not included because of language restrictions, overall, there were no major concerns.  Findings are likely to be reliable.  Concerns highlighted in review were appropriately considered in conclusions.  Conclusions were supported by the evidence and included consideration of the relevance of included studies. | Concerns identified in 2 domains (eligibility and selection criteria) were rated as high and were not addressed in the interpretation of findings.  The review did not adhere to its predefined objective. There was also the potential risk that some studies were not included because of language restrictions.  Limitations of the included studies were highlighted and considered. |

Supplementary Table 4 Assessing certainty of evidence for lower versus higher carbohydrate diets on glycated haemoglobin (HbA1c) in the longer-term (≥12 months) using the GRADE approach

| **Meta-analysis** | Sainsbury et al^(6)^ (12 RCTs, n=1403) |
| --- | --- |
| **Results of meta-analysis**  (mean difference in change, %) | No statistically significant difference in effect between lower and higher carbohydrate groups.  -0.09 (-0.21, 0.03), p=0.12 |
| **Domains for assessing certainty of evidence:** | |
| - risk of bias | Half (6/12) of the RCTs in meta-analysis were at unclear or high risk of bias.  Sensitivity analysis after exclusion of 1 RCT at high risk of bias: greater reduction in HbA1c with the lower carbohydrate diet: -0.13 (-0.26, -0.01), p=not reported  Some concerns - potential limitations are likely to lower confidence in the estimate of effect |
| - imprecision | Large number of studies in meta-analysis and large sample size |
| - inconsistency | I^2^=30% (moderate heterogeneity)  Overlap of confidence intervals; results generally in same direction (8 out of 12 RCTs) |
| - indirectness | Studies were conducted in population of interest |
| - publication bias | No evidence of publication bias (Eggers test) |
| **Strength of evidence** | **MODERATE**   - Downgraded by 1 level for risk of bias |
| **Difference in effect/**  **Strength of evidence** | **No difference in effect/MODERATE certainty** |

Supplementary Table 5 Assessing certainty of evidence for lower versus higher carbohydrate diets on glycated haemoglobin (HbA1c) in the longer-term (≥12 months) using the USDA/DGAC^(15)^ approach

| **Meta-analysis** | Sainsbury et al^(6)^ (12 RCTs, n=1403) | |
| --- | --- | --- |
| **Results of meta-analysis**  (mean difference in change, %) | No difference in effect between lower and higher carbohydrate groups.  -0.09 (-0.21, 0.03), p=0.12 | |
| **Grading elements for consideration:** | | **Grade** |
| **Risk of bias** | 6/12 RCTs: unclear or high risk of bias. | Moderate |
| **Consistency**  Consider degree of similarity in direction and magnitude of effect across body of evidence | I^2^=30%  Overlap of confidence intervals; results generally in same direction (8 out of 12 RCTs). | Moderate |
| **Directness**  Occurs when following are directly related to the systematic review question:   - populations - intervention - comparators - outcomes of interest | - populations directly related to systematic review question - Intervention diets – lower carbohydrate diet definition varied across studies - Comparator diets varied widely across studies - Outcomes of interest were related to SR question | Moderate |
| **Precision**  consider:   - sample size - number of studies - variability within and across studies | - sample size of individual studies ranged between n=61 to 419; total sample size n=1403 - Large number of studies (12 RCTs) | Strong |
| **Generalizability**  Consider if findings applicable to population of interest (adults with type 2 diabetes in the UK) | Participants   - adults living with type 2 diabetes and overweight/obesity - from predominantly white populations in high income countries   Findings may not be applicable to adults with type 2 diabetes without overweight/obesity or to adults with type 2 diabetes of different ethnicities | Moderate |
| **Overall grade:** Above assessments are used to facilitate discussion and selection of an overall grade. | | **Moderate** |
| **Difference in effect/**  **Strength of evidence** | **No difference in effect/MODERATE certainty** | |
